# Impact of Ocean Warming on Tropical Cyclone Size and Its Destructiveness

**DOI:** 10.1038/s41598-017-08533-6

**Published:** 2017-08-15

**Authors:** Yuan Sun, Zhong Zhong, Tim Li, Lan Yi, Yijia Hu, Hongchao Wan, Haishan Chen, Qianfeng Liao, Chen Ma, Qihua Li

**Affiliations:** 1 Key Laboratory of Meteorological Disaster, Ministry of Education (KLME)/Joint International Research Laboratory of Climate and Environmental Change (ILCEC)/Collaborative Innovation Center on Forecast and Evaluation of Meteorological Disasters (CIC-FEMD), Nanjing University of Information Science and Technology, Nanjing, 210044 China; 2 College of Meteorology and Oceanography, National University of Defense Technology, Nanjing, 211101 China; 3 IPRC and Department of Atmospheric Sciences, University of Hawaii at Manoa, Honolulu, Hawaii 96815 USA; 4 Chinese Academy of Meteorological Sciences/Chinese Meteorological Society, Beijing, 100081 China; 5 Meteorological Observatory of Hankou Station, Wuhan, 430000 China

## Abstract

The response of tropical cyclone (TC) destructive potential to global warming is an open issue. A number of previous studies have ignored the effect of TC size change in the context of global warming, which resulted in a significant underestimation of the TC destructive potential. The lack of reliable and consistent historical data on TC size limits the confident estimation of the linkage between the observed trend in TC size and that in sea surface temperature (SST) under the background of global climate warming. A regional atmospheric model is used in the present study to investigate the response of TC size and TC destructive potential to increases in SST. The results show that a large-scale ocean warming can lead to not only TC intensification but also TC expansion. The TC size increase in response to the ocean warming is possibly attributed to the increase in atmospheric convective instability in the TC outer region below the middle troposphere, which facilitates the local development of grid-scale ascending motion, low-level convergence and the acceleration of tangential winds. The numerical results indicate that TCs will become stronger, larger, and unexpectedly more destructive under global warming.

## Introduction

Tropical cyclones (TCs) are among the most destructive natural disasters on Earth^1, 2^. Hazards associated with TCs are most commonly found over the western North Pacific and the North Atlantic^3, 4^. An improved understanding of the factors that control TC activity (e.g., frequency, track, intensification, and destructiveness) is needed, especially in the context of global warming^3, 5–10^. Sea surface temperatures (SSTs) in most regions of TC formation have increased by several tenths of a degree Celsius during the past several decades^11^. Since the ocean is the main source of energy supply for TC intensification^12–17^, changes in ocean environments can contribute greatly to changes in the TC activity^6, 18–25^. While theoretical arguments and modelling studies have suggested that the TC intensity should increase with increased SST^26–29^, debates on the future projection of TC destructiveness still exist^3, 24, 30, 31^. Moreover, some physical properties, such as the change in TC size, which is critical for assessing the destructive potential of TCs under global warming, have been unfortunately omitted in previous studies. Due to the lack of storm size information in historical data, many previous studies often assumed a fixed storm dimension in estimating TC destructiveness^3^. However, this assumption may not hold in the future under the global warming scenario because storm size is sensitive to changes in SST, which has been shown clearly in recent modeling studies^5, 17, 32, 33^. In this study, we focus on understanding how storm size and TC destructive potential might change under various ocean-warming scenarios.

The large natural variability in a relatively short observational period makes it difficult to determine what percentage of the observed TC activity changes can be attributed to the ocean warming due to forcing by greenhouse gases^23, 24^. In this study, we try to eliminate the interference of natural variability and shed new light on this topic. A series of numerical experiments on the sensitivity of TC size to SST changes have been conducted with a focus on the change of TC size and its impact on TC destructive potential. Due to the inherent limitation of numerical models in use, the simulated TC activities (e.g., the TC frequency, duration, intensity, size and track) may differ from the observations at various degrees from slightly to radically. Although the simulated TC track and intensity have a certain errors, such errors do not necessarily affect the sensitivity of the model TC size response to SST warming. The fact that both the course-resolution climate simulations and higher-resolution case simulations show consistent and robust TC size change signals implies that the current model result is reliable. The Weather Research and Forecasting (WRF) model, which was designed to meet both the operational forecasting and atmospheric research needs, was developed by the National Center for Atmospheric Research (NCAR) and the National Centers for Environmental Prediction (NCEP) in the U. S. WRF is applied in the present study for both climate simulations and TC case simulations to comprehensively investigate the sensitivity of TC activity to SST change. Results from the long-term climate simulations enable us to discover the universal phenomena, while results from the high-resolution TC case simulations are helpful for us to reveal the corresponding mechanisms. The data used in this study, the model configuration and the experimental design are described in the section of Methods.

## Results

### PDS vs PDI

The TC destructive potential is a better indicator of TC threat than the storm intensity or frequency^3^. The Power Dissipation Index (PDI) proposed by Emanuel^3^ in 2005 is widely used to estimate the destructive potential of TC^24, 25, 34^. The PDI of a TC is defined as the sum of the cube of the maximum wind speed (MWS) during the lifetime of the TC, in which the change in the TC size is ignored. However, the TC size determines the total storm area where the 10-m sustained winds exceed the TC intensity (>17 m s^−1^), i.e., the area of gale-force winds (AGW), which is an important damage potential parameter^33, 35^. Thereby, despite its wide usage, the PDI is not a perfect and all-inclusive measurement of the TC destructive potential due to the lack of information on the storm size^36^.

In order to quantitatively illustrate the limitation of PDI, the storm size-dependent TC destructive potential (PDS) is calculated. Our strategy is to analyze the long-term changes in TC size and TC activity based on WRF simulations under the present climate scenario but with different SST warming conditions/designs over the western North Pacific (WNP) and the North Atlantic (NA). Correspondingly, one set of control experiments (CTRL) and two sets of sensitivity experiments (E_FSST_, E_ΔSST_) are conducted. In the CTRL, the realistic and unchanged SST pattern is used. In the E_FSST_, the SST represents a future pattern and is obtained by superimposing the future SST anomalies on the SST in CTRL. The projected SST anomalies are identical to that in Lau *et al*.^33^ (Fig. S1, Supplementary Information). In the E_ΔSST_, the uniform SST warming of 1 °C and 2 °C is assumed respectively for the entire simulation domain (i.e., E_all+1_ and E_all+2_). Details on the data and model simulations can be found in the Methods section. Model outputs are analyzed to investigate how the TC size would change under the ocean warming and how these changes may impact the TC PDS.

The PDS, which is calculated over an area occupied by gale-force (>17 m s^−1^) winds near the TC core and over the TC lifetime, is given by:

1 $${\rm{PDS}}={\int }_{0}^{\tau }{\int }_{0}^{{A}_{0}}{C}_{D}\rho {|{\bf{V}}|}^{3}{\rm{d}}A{\rm{d}}t$$

where *ρ* is the surface air density, *C*_*D*_ is the surface drag coefficient, |**V**| is the magnitude of the surface wind at 10 m, *A*_0_ is the area of gale-force winds, and *τ* is the lifetime of TC. The PDS reflects the total power of gale-force winds dissipated by the TC over its lifetime.

**Figure 1:**
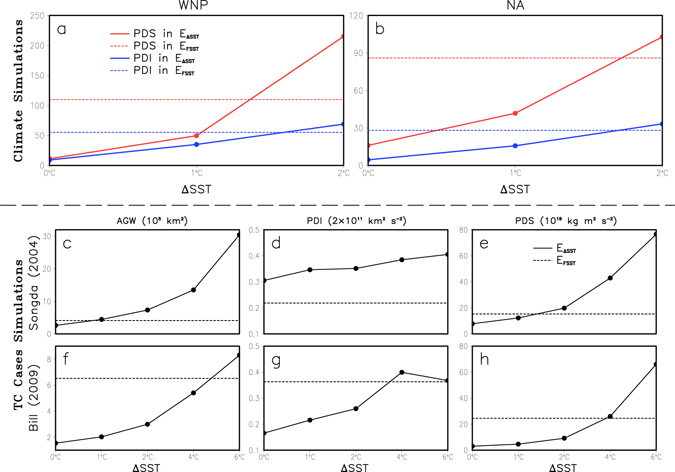
Dependence of the TC destructive potential on SST warming. (**a**,**b**) Sensitivity of annual PDI (2 × 10^11^ m^3^ s^−2^) and PDS (10^18^ kg m^2^ s^−2^) during the typhoon season (May-November) to SST warming over the WNP and the NA in the climate simulations. The annual PDI and PDS are averaged over 10 years (2001–2010). (**c**–**h**) Changes in the time-averaged AGW, PDI, and PDS in response to increased magnitude of SST warming and future SST scenario simulated by the sensitivity experiments for the TC cases of Songda (2004) in the WNP and Bill (2009) in the NA. (E_ΔSST_ includes the CTRL, E_all+1_, and E_all+2_).

Figure 1a and b show the sensitivity of the annual PDI and PDS to increases in SST over the WNP and the NA in the climate simulation. The annual PDI and PDS are the sum for all the TC cases during the typhoon season in a year (i.e., May-November) averaged over 10 years (2001–2010). Following the increases in SST in each case, the calculated PDI and PDS both increase significantly at the 0.05 level of significance by the Welch’s t-test (Table S1). The comparison between results of CTRL and E_FSST_ shows that with the increase in SST caused by double CO_2_, the PDS increases by 883.2% and 434.6% in the WNP and NA respectively, which are much greater than that of PDI (285.1% and 190.0%) (Table S2). Further analysis indicates that within the range of SST change from 0 °C to 2 °C, the PDS grows much more rapid than the PDI (Fig. 1a and b). Specifically, as the SST increases one degree Celsius, the PDS in the WNP and the NA can increase by about 340% and 150% respectively. This result indicates that the estimated increase in the TC destructive potential under global warming would be severely underestimated if changes in the storm size were not taken into account.

The calculated 10-year (2001–2010) averaged TC numbers in the CTRL are basically consistent with the observations (26.5 vs 29.9 in the WNP and 15.7 vs 17.4 in the NA), indicating that the WRF model is capable of reproducing TC activities over the WNP and the NA. However, the PDI calculated from the model output (i.e., CTRL) is smaller than that calculated by Emanuel^3^ using the best track data from the Joint Typhoon Warning Center (JTWC). This is probably due to the relatively coarse spatial resolution (20 km) used in the long-term climate simulation. Note that it is not necessary to precisely reproduce the TC activities over the WNP and the NA in terms of TC intensity. Our conclusions are more dependent on the comparisons among the sensitivity experiments and less dependent on the accuracy of the CTRL results. Furthermore, the results of high spatial resolution (4 km) runs with selected TC cases also present a similar conclusion that the TC size should be considered in the calculation of TC destructiveness. This issue will be further discussed later.

### Four contributing factors for PDS

As indicated in Eqs (1) and (2) in Emanuel^3^, the PDI is determined by TC frequency (annual TC number *N*), intensity (maximum wind speed *I*) and duration (lifetime *L*) and the PDS [i.e., PD in his Eq. (1)] is affected by one extra factor, i.e. the TC size (area of the gale-force winds *S*), in addition to the three factors mentioned above. Similar to the method proposed by Emanuel^22^, we estimate the contribution of each factor to the PDS in the WNP and the NA respectively (Table 1). Apparently, the TC size dominates other factors in the increase of PDS for both the WNP and the NA basins. This is consistent with the model results of Lau *et al*.^33^, which indicated that the size of the super storm Sandy increases significantly with a warmer SST. In general TC size does not depend on RMW or MWS. However, for the climate simulations with varying SST over the WNP and the NA, the outer slope of azimuthal mean wind speed is quite similar (Fig. S2, Supplementary Information), and as a result, the TC size (AGW) is somehow related to RMW and MWS.. In this study, due to the increases in both the RMW and MWS, the calculated TC size increases significantly following the increases in SST over the WNP and the NA (Table S2 and Fig. S2, Supplementary Information). More interesting, the TC size effect on the PDS shows an increasing trend when we compare the results from the E_all+1_ with that from the E_all+2_ in the WNP and the NA, while the effects of other factors (such as the TC frequency and duration) show an opposite or unchanged trend (Table 1). This implies that with a continuous warming of global SST, the TC size effect on the PDS will increase. The overall analysis indicates that the change in TC size under a warmer ocean condition is a major contributor to the increase in the PDS. Thus, it cannot be ignored in estimating the TC destructive potential under global warming.

**Table 1:** Contributing factors to 10-year (2001–2010) averaged PDS change. Contributions of the TC case number (*N*), lifetime (*L*), intensity (*I*) and size (*S*) to the PDS changes in the SST sensitivity experiments (E_FSST_, E_all+1_, and E_all+2_) compared to that in the CTRL experiments over the WNP and NA.

Factors	Basin					
	WNP			NA		
	E_FSST_	E_all+1_	E_all+2_	E_FSST_	E_all+1_	E_all+2_
*N* (%)	19.9	24.6	12.7	45.7	53.3	40.7
*L* (%)	8.0	9.0	9.3	8.6	18.7	12.2
*I* (%)	32.9	34.7	33.5	15.2	10.4	16.9
*S* (%)	39.2	31.8	44.4	30.5	17.5	30.1

### Mechanism for TC size expansion

In order to investigate the causes for the increase in the storm size under global warming, we conducted a suite of high-resolution (4-km horizontal resolution) sensitivity experiments for two TC cases, i.e. Songda (2004) in the WNP and Bill (2009) in the NA. It is expected that the numerical simulations with higher resolution can reproduce more realistic responses of the TC size, intensity, structure and destructiveness to SST warming^37, 38^. Similar to the model design in the aforementioned climate simulations, three experiments with different SST patterns (i.e., CTRL, E_FSST_, E_ΔSST_) are conducted. Figure 1c–h show the changes in time-averaged AGW, PDI, and PDS calculated from the sensitivity experiments with increased magnitude (from 0 to 2 °C) of SST warming (E_all+0_/CTRL, E_all+1_, and E_all+2_) and with the future SST (E_FSST_). The comparison of results of the CTRL and the E_ΔSST_ indicates that all the aforementioned variables monotonically increase with the magnitude of SST warming (i. e., 0, 1, and 2 °C). Meanwhile, the increase in the PDS is significantly greater than that in the PDI. This result is consistent with the results of our climate simulations.

It is worth noting that the impact of SST warming on the TC activity not only is related to the magnitude of SST change, but also associated with the SST change pattern^5^. In this study, the domain-averaged increase in the future SST in E_FSST_ is 1.86 °C in the Songda case and 3.55 °C in the Bill case. As expected, for the Bill case, the calculated variables in E_FSST_ are all greater than those in E_all+2_ due to the larger magnitude of SST warming in E_FSST_. However, for the Songda case, the calculated variables in E_FSST_ are significantly smaller than those in E_all+1,_ except for the AGW. This result suggests that in addition to the magnitude of SST warming, the pattern of SST warming also plays an important role in determining the TC activities. Further analysis indicates that for Songda case, the weak time-averaged TC intensity in E_FSST_ can be attributed to the early northward turning of the simulated TC (Fig. S3, Supplementary Information). Specifically, at the early stage of Songda in E_FSST_, the simulated TC intensity increases quickly and reaches its lifetime peak intensity (900.5 hPa and 59.8 m s^−1^ in terms of MSLP and MWS, respectively) at about 0000 UTC 3 September 2004, which is even stronger than that in E_all+2_ (Fig. S4, Supplementary Information). Nevertheless, the TC intensity in E_FSST_ subsequently decreases quickly with the MSLP of 1010.8 hPa and MWS of 18.0 m s^−1^ at 0000 UTC 6 September 2004. Such a rapid weakening of the TC is attributed to the early northward turning of the TC in E_FSST_ (Fig. S3, Supplementary Information). At 0000 UTC 3 September, the simulated TC in E_FSST_ has arrived at the latitude of 31.1°N, where the cold ocean is not conductive to further intensification of the TC. However, compared with that in Songda case, the impact of SST warming on the TC track is much weaker in Bill case (Fig. S5, Supplementary Information). It seems that the SST warming may have a significant impact on the TC track over the WNP, at least for the Songda case. As suggested by Sun *et al*.^39^, a TC with a larger size tends to turn northward earlier, which is a result of the interaction between the TC and the western Pacific subtropical high over the WNP. Thereby, in E_FSST_, the large TC size (AGW) at early stage (from 1800 UTC 31 August to 0000 UTC 2 September 2004) of the TC lifetime may be an important reason for the northward turning of TC in Songda case (Figs S3 and S4c, Supplementary Information). More importantly, for both the Songda and Bill cases, during initial 4 days TC tracks are quite similar among all sensitivity experiments, but TC size has shown a clear difference (Figs S3,S4 and S5, Supplementary Information). This suggests that TC size is primarily affected by SST warming induced atmospheric condition changes, not due to TC position difference induced environmental changes.

**Figure 2:**
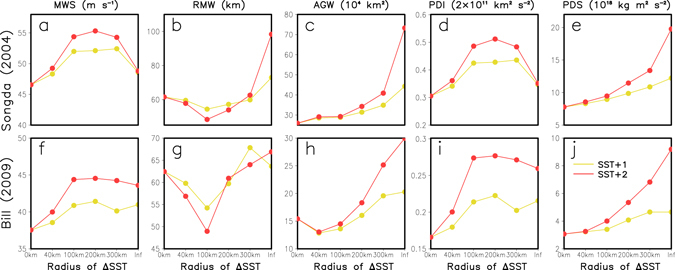
Changes in time-averaged MWS, RMW, AGW, PDI and PDS with the radial extent of the warm SST pool in the sensitivity experiments. (**a–e**) Songda (2004); (**f–j**) Bill (2009).

To further investigate the impact of SST pattern (e.g., inner and outer SST warming) on TC activities, a number of sensitivity experiments with the SST warming occurring within a specified radius of the TC center were conducted. For example, E_100+2_ represents an experiment with a SST warming of 2 °C within a radius of 100 km of the TC center (details can be found in the section of Methods). Figure 2 presents the changes in the time-averaged MWS, RMW, AGW, PDI, and PDS calculated from E_ΔSST_. Results of Songda and Bill simulations indicate that the MWS, RMW, and PDI do not monotonically change with the increased radial extent of SST warming, whereas the AGW and PDS do. There is a critical radius (CR) for the MWS, PDI, and RMW. Within a range of about 100 km, the MWS and the PDI increase with an increased radius of SST warming, while beyond this range, the MWS and the PDI decrease or remain unchanged as the radius of SST warming further increases. The RMW, on the other hand, behaves opposite. In addition to the PDS, the AGW is also determined by both the MWS and the RMW (Fig. S2, Supplementary Information). Under the joint effects of the MWS and the RMW, the AGW and PDS increase with an increasing radius of SST warming, except that the PDS decreases when the SST warming radius increases from 300 km (E_300+_) to infinite (E_all+_) in Songda case.

The above numerical results indicate that the effect of inner SST warming and that of the outer SST warming are opposite in determining the changes in the MWS and the RMW. It appears that the TC intensity change is more sensitive to the inner SST warming (Fig. 2a and f) while the TC size change is more sensitive to the outer SST warming (Fig. 2c and h). Thereby, it is the joint effect of the inner and outer SST warming that determines the final changes in the TC intensity and TC size, and thus the TC destructive potential under large-scale ocean warming. This mechanism can explain why the simulated TC size in E_FSST_ is larger than that in other experiments in the early stage of the TC for the cases of both Songda and Bill. For the Songda case, as is shown in Fig. S3 (Supplementary Information), the SST warming in E_FSST_ is not homogenous but increases with the latitude. At the early period (before 0000 UTC 3 September), the large SST warming in the north of the model domain occupies the outer region of the TC, which results in a stronger outer SST warming that contributes to the larger TC size in E_FSST_ (Figs S3 and S4c, Supplementary Information). This is also the reason why the simulated TC size is large from 0000 UTC 19 August to 0000 UTC 23 August 2009 in E_FSST_ for Bill case (Figs S4f and S4, Supplementary Information). For the TCs over the WNP, as suggested by Sun *et al*.^39^, a larger TC size can lead to earlier northward turning of the TC. However, this finding may not apply to the TCs over the NA (e.g., Bill case). More details on the impact of SST warming on TC track will be addressed in our next work.

Results of both the climate simulations and the simulations of TC cases show that, the joint effect of the inner and outer SST warming leads to the increases in TC intensity and TC size and also a larger PDS (Figs 1 and 2, and Figs S2 and S6 in Supplementary Information). Therefore, it is deduced that the TC will become not only stronger (e.g., MWS) but also larger (e.g., AGW) in the context of global warming, resulting in a much stronger TC destructive potential (e.g., PDS). It is relatively easy to understand that the TC intensity will increase under the ocean warming since the surface entropy flux (SEF) will increase in response to the ocean warming^5^. However, what is the reason for the TC size change? Why does the TC size increase under the large-scale ocean warming?

**Figure 3:**
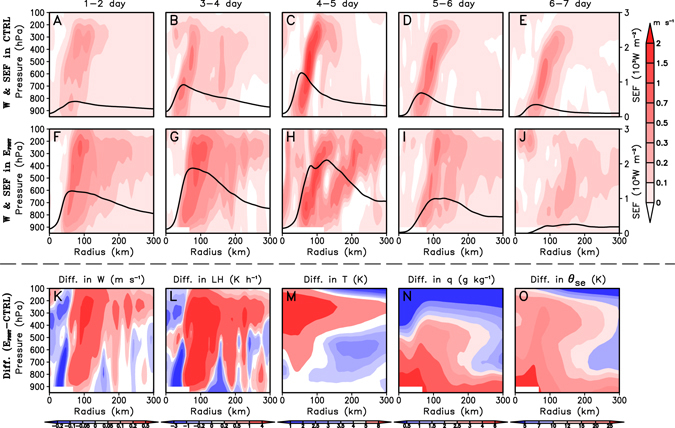
Impacts of SST warming on the time- and azimuthal-averaged TC structure for Bill (2009) case. (**a**–**j**) Radial**-**cross sections of the vertical velocity (shaded; m s^−1^) and surface entropy flux (contours; 10^3^ W m^−2^) in the CTRL and E_FSST_ at various one-day average periods; (**k**–**o**) Radial-cross sections of the 1–2-day averaged differences in the vertical velocity (m s^−1^), latent heating (LH; K h^−1^), temperature (*T*; K), water vapor mixing ratio (*q*; g kg^−1^) and potential pseudo-equivalent temperature (*θ*_se_; K) between E_FSST_ and CTRL experiments.

In order to answer this question, we examine the evolution of the vertical velocity and the SEF in the CTRL experiment and in the E_FSST_ for Bill (2009) case (Fig. 3a–j). In the CTRL, the convection and the SEF show an enhanced trend with the time, but there is no substantial change in the eyewall radius (approximately RMW) as the convection and the SEF are largely confined within a 70-km radius of the TC center (Fig. 3a–e). Compared with those in the CTRL, temporal changes in the vertical velocity and the SEF in the E_FSST_ are more complicated (Fig. 3f–j). In the E_FSST_, during the early developing stage of Bill, convection and the SEF are mostly confined to a relatively narrow region in the eyewall area with a radial distance of about 50-km from the TC center (Fig. 3f). The intensity of convection near the eyewall increases gradually with time due to the enhanced inner-SEF caused by the inner SST warming. On the other hand, the SEF also increases in the TC outer region as a result of the outer SST warming (Fig. 3g). This contributes to the intensification of convection in the outer region by increasing the atmospheric instability in this region, which will be illustrated by comparing the E_FSST_ results with the CTRL results in the next paragraph.

As suggested by Sun *et al*.^40, 41^, enhanced eyewall convection caused by the inner SST warming could strengthen the upper-level anvil clouds and promote their outward extension, as shown in Fig. 3k. The anvil clouds can warm the upper-troposphere (around 450–150 hPa) due to the water vapor condensation in the clouds, while the anvil showers falling from the clouds cool the middle-troposphere (around 700–450 hPa) due to the evaporative cooling in the outer region of the TC (Fig. 3l and m). Meanwhile, the compensating subsidence driven by the enhanced eyewall convection leads to a dry lower troposphere (around 800–700 hPa) through dry advection (Fig. 3n). In fact, without the effect of the outer SST warming, such a temperature and moisture profile in the TC outer region will make the atmosphere more stable, and thus impedes the development of convection in the outer region of TC. This is the reason why the inner SST warming can cause decreases in the RMW (see Fig. 2b and g). However, the outer SST warming makes the situation substantially different. Higher SST in the outer TC region directly contributes to the warming and moistening in the lower-troposphere (below 850 hPa) through the heat and moisture transport from the ocean to the atmosphere (Fig. 3m and n). As a result, the increase of the pseudo-equivalent potential temperature (*θ*_se_) increases in the lower troposphere is much more than that in the middle troposphere (Fig. 3o). This leads to the establishment of a convectively unstable stratification below the middle troposphere that is favorable for the development of the outer spiral rainbands in the TC outer region. The development of outer spiral rainbands outside the TC eyewall eventually leads to the increases in RMW and thus TC size (Fig. 2g and h), which is stated in detail in the following paragraph.

The rainbands-related peripheral convection in the outer region (within the range of 1–3 times RMW) induces low-level convergence, which leads to an acceleration of inflow outside of the radius and a deceleration of inflow inside of the radius (Fig. S7d, Supplementary Information). As suggested by Xu and Wang^32^, the acceleration of inflow results in the strengthening of local tangential flows, which further facilitates the growth of SEF (Fig. 3h and Fig. S6d in the Supplementary Information). The increased SEF in turn promotes the development of large-scale convection. Apparently there exists a positive feedback loop among convection, low-level wind and the SEF in the outer region, which favors the expansion of the gale-wind radius and thus the increase of TC size (see Fig. 3f–j and Fig. S6d in the Supplementary Information).Similar results can be found in the Songda (2004) case (Figs S6a,b, S7a,b, and S8, Supplementary Information).

Due to its coarse resolution, the climate model cannot well reproduce the fine-scale TC core structures and narrow spiral rainbands in the outer region. Nevertheless, the major mechanism that causes the increase of TC size as SST increases appears to be the same in both the 20-km climate simulations and 4-km higher-resolution simulations. For example, a set-up of convective unstable stratification in the outer region is well captured in the 20-km resolution simulations. Take the comparison between E_all+1_ and CTRL for example, as the underlying SST increases, the equivalent potential temperature (*θ*_se_) increases near the surface (due to the SST warming) and decreases in the middle troposphere due to rain-drop induced evaporation as well as dry advection (Fig. S9, Supplementary Information), which is consistent with the results in the high-resolution TC simulations (see Fig. 3o). This *θ*_se_ profile leads to the establishment of a convectively unstable stratification favorable for the development of grid-scale ascending motion in the outer region. The ascending motion induces low-level convergence, which eventually leads to the accelaration of tangentail wind outside of the convection region and causes the increase of TC size.

## Discussion

**Figure 4:**
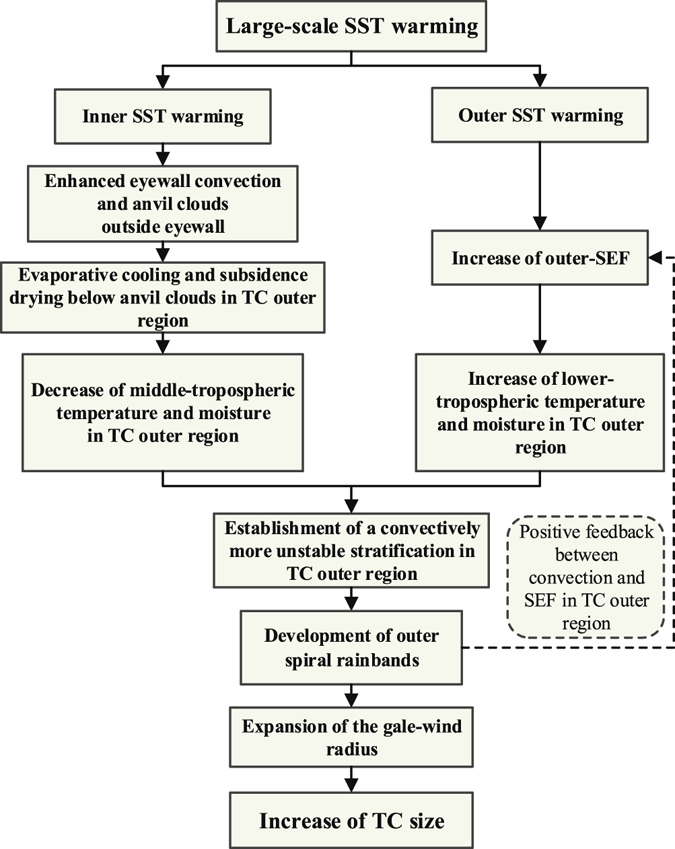
Schematic diagram summarizing the possible mechanisms responsible for the impact of large-scale SST warming on TC size.

The climate simulation results in this study have indicated that the estimated TC destructive potential in the context of global warming would be severely underestimated if the change in the storm size were not taken into account. Further analyses of the results from the simulations of the selected TC cases have revealed that the impact of SST warming on TC activities is related to both the magnitude and the pattern of SST change. Specifically, the effects of the inner and outer SST warming on the MWS and RMW are opposite. The increase in inner (outer) SST can lead to increases (decreases) in the MWS but decreases (increases) in the RMW. Under the joint effects of inner and outer SST warming, TCs become not only stronger (e.g., MWS) but also larger (e.g., AGW). As a result, the TC destructive potential will become greater (e.g., PDS). The physical mechanisms responsible for the effect of large-scale SST warming on the TC size are identified through a comprehensive analysis that is schematically summarized in Fig. 4. In addition, besides the SST, the ocean heat content also plays a role in regulating the TC intensity, in particular for slowly moving TCs. Investigating its effect requires the long-term simulation of a coupled ocean-atmosphere model. To the first order, we simply in the current study neglect this effect, and focus on the investigation of the effect of increasing SST on the change of TC size and destructive potential (PDS).

It has been shown by previous studies that SST warming may significantly modify the upper tropospheric temperature and thus atmospheric static stability^8, 34^. To investigate the possible impact of atmospheric lateral boundary condition on the model results, we have conducted three extra sets of 5-month (May-November) ensemble simulations with lateral boundary conditions from global model simulations with different SST warming patterns (i.e., CTRL_extra_, E_all+1-extra_, and E_all+2-extra_). More details on the model design of the extra climate simulations can be found in the Supplementary Information. To see how different the simulated upper-tropospheric temperature fields are between original and new sets of simulations, we examine area-averaged 200-hPa temperature difference between the SST sensitivity experiments and the CTRL experiment averaged from May to November in both sets of experiments. Note that in both the cases, the simulated upper-tropospheric temperature increases remarkably with the increase of underlying SST, regardless of lateral boundary conditions posed (Fig. S10, Supplementary Information). This implies that even in the original simulations with specified present-day lateral boundary conditions, the interior atmospheric conditions are still primarily affected by the SST effect. More importantly, the model results of extra simulations also indicate that, the increase of PDS are much greater than that of PDI due to the large increase of storm size (Table S5, Supplementary Information). This further validates our main conclusion that the estimated increase in the TC destructive potential under ocean warming would be severely underestimated if changes in the storm size were not taken into account.

Our results have important implications for changes in TC activity (e.g., TC intensity, size, and destructiveness) under global warming. However, TC will not become stronger, larger, and thus more powerful in the near future because of the recent global-warming hiatus^42^. The change in global SST in a short period may have large uncertainties as it is a quite complex issue, but global SST is expected to continuously increase in the long-term according to the projected multi-model mean SST anomalies from 33 CMIP5 models for doubled-CO_2_ experiments^33^. Thereby, although the recent global-warming hiatus may bring some uncertainties to short-term global SST changes in the near future, we believe that it should not change our main conclusions. The strengthened TC destructive potential poses a heightened threat to human society and terrestrial/marine ecosystems^43^.

Finally, multiple model simulations are needed to confirm the results of the present study since the results may be model-dependent. Additionally, large-scale circulation could change under the ocean warming. Changes in the lateral boundary forcing related to large-scale circulation and the coupled SST effects are not considered in the present study. It would be interesting to know how these effects would modify the TC size, which will be the focus in our next research.

## Methods

### Data

The National Centers for Environmental Prediction (NCEP) 1° × 1° global final (FNL) analysis data at 6-hour intervals for the time period of 10 years (2001–2010) is used to provide the initial and lateral boundary conditions for the model experiments of both climate simulations and TC cases simulations. SST is updated using the NCEP-FNL analysis data. A variety of observational data are assimilated into the NCEP-FNL analysis product to ensure its reliability. These data have been widely used in atmospheric studies. The NCEP-FNL analysis data are available at the Research Data Archive (http://rda.ucar.edu) in dataset number ds083.2.

### Model simulations

The Weather Research and Forecasting Model (Version 3.3) with Advanced Research dynamical core (WRF-ARW, codes available at http://www.wrf-model.org) is used to conduct two suites of experiments: long-term climate simulations and high-resolution TC cases simulations. Dimensions of the model domains, the time span of the model integration, and the selected physics schemes are listed in Table S3.

The climate simulations, with a 20-km spatial resolution, are conducted to investigate the response of TC activity, especially the TC size and TC destructive potential, to increases in SST. The sensitivity experiments cover the tropical cyclone seasons (from May 1 to November 1) over a 10-year period (2001–2010) in the western North Pacific (100° to 180°E, 0° to 60°N) and the North Atlantic (−100° to −20°W, 0° to 60°N). Three sets of 5-month (May-November), 10-member (2001–2010) ensemble numerical experiments with the same atmospheric initial and lateral boundary conditions but different SST patterns were carried out. In the CTRL experiments, SST remains unchanged; in the sensitivity experiments (E_FSST_, E_ΔSST_), SST is increased. In the sensitivity experiments, similar to that in Lau *et al*.^33^, the projected multi-model mean SST anomalies from the doubled-CO_2_ experiments of 33 models of the Coupled Model Intercomparison Project (CMIP5, see http://cmip-pcmdi.llnl.gov/cmip5/ for more details) were superimposed on the SST field in the CTRL experiments to obtain the future SST scenario for the E_FSST_ experiments. Increases in SST in the E_all+1_ and E_all+2_ are homogeneously set to 1 and 2 °C in the entire model domain, respectively. For example, E_all+2_ represents an experiment with a uniform SST warming of 2 °C for the entire simulation domain. Note that, due to the relatively high spatial resolution (20-km) and frequent output (6-hr intervals) of the model, the computation and storage space required for the climate simulations in this study are huge (about 300,000 machine hours and 16 TB space). This is the reason why the climate simulations didn’t cover a longer time span and over a larger domains. However, this does not affect the reliability of the main conclusions of the present study, since the climate simulation results are robust in this study.

The TC-case experiments are designed to simulate two representative TC cases (i.e., Songda 2004 in the WNP and Bill 2009 in the NA) to investigate the response of TC activity to increased SST over different radial extents and thus to disentangle the mechanism for the TC size expansion. The model domains for the two TC cases are doubly nested, and the two-way interactive feedback is activated between the 20 km-resolution outer domain and the 4 km-resolution inner domain. The inner domain, which is designed to define the TC, automatically moves to follow the positions of the storm via the automatic vortex-following algorithm^44^. Similar to the design of our climate experiments, we have also conducted experiments with the present SST and future SST scenario (i.e., CTRL and E_FSST_) for the cases of Songda and Bill. Moreover, to investigate the sensitivity of TC activity to not only the magnitude but also the pattern of SST change, an additional set of sensitivity experiments with varying magnitude (i.e., 1 and 2 °C) and radius (i.e., 40-, 100-, 200, and 300-km) of SST warming are conducted. Specifically, the SST is increased according to a weighting function that it is maximized within a prescribed radius of the TC center, and then linearly decreases outward to zero beyond the prescribed radius plus 20 km and remains zero farther outward. Note that, since the TC is not static, the SST field is modified on the basis of the NCEP-FNL SST data at each time step to ensure that the center of the changed SST region is always consistent with the TC center in our sensitivity experiments. The summary of the sensitivity experiments for the selected TC cases are listed in table S4. The extent of the outer domain and the simulated TC track in some representative experiments are shown in Figs S3 and S5 (Supplementary Information) for Songda and Bill cases, respectively. Because of the heavy burden of computation and storage (about 10,000 machine hours and 1 TB for each TC case), we only select a single TC case for each ocean basin (i.e. Songda in the WNP and Bill in the NA) in this study. The robust results of the two TC cases are sufficient for us to reach the major conclusions regarding the impact of ocean warming on the TC size and TC destructive potential. We believe that the findings of this study can also apply to other TC cases.

### Vortex detection criteria

In the model, a TC is identified to be a vortex according to thermodynamic and dynamic criteria. In this study, several criteria are chosen so that the TC genesis frequency in each year is closest to the observation. These criteria are:

1. The maximum relative vorticity at 850 hPa exceeds 4.0 × 10^−5^ s^−1^.
2. The grid point with the minimum sea-level pressure within a radius of 500 km from the position of the maximum relative vorticity is defined as the center of the TC.
3. The maximum wind speed at 10 m within a radius of 600 km from the TC center is greater 12 m s^−1^, and the lifetime maximum wind speed at 10 m is greater than 17 m s^−1^.
4. Temperature at 300 hPa is 1 °C higher than the area-averaged temperature within a radius of 400 km from the TC center.
5. TC lifetime must be at least 48 hours.
6. The genesis location is located to the south of 40°N over the ocean.

A sensitivity test with use of different vortex detection criteria indicates that the main conclusion that PDS increases at a much faster rate than PDI as the SST increases remains unchanged, even though the TC number may change somewhat (Figs S11 and S12, Supplementary Information).

### PDS analysis

Emanuel^22^ provided a useful way to estimate the contribution of each of the three factors (i.e., TC frequency, intensity, and duration) to the PDI. The PDS is affected not only by those three PDI-contributing factors but also by the TC size. Based on the methodology proposed by Emanuel^22^, contributions of the TC lifetime, intensity, and size to the change in PDS can be estimated by:

2 $${L}_{i}=\frac{{\int }_{0}^{\tau }{V}_{{\rm{\max }}}{\rm{d}}t}{{V}_{{\rm{smax}}}},\,L=\frac{1}{N}\sum _{1}^{N}{L}_{i}=\frac{1}{N}\sum _{1}^{N}\frac{{\int }_{0}^{\tau }{V}_{{\rm{\max }}}{\rm{d}}t}{{V}_{{\rm{smax}}}}$$

3 $$I=\frac{\sum _{1}^{N}{\int }_{0}^{\tau }{V}_{\max }^{3}dt}{\sum _{1}^{N}{L}_{i}}$$

4 $$S=\frac{{\rm{PDS}}}{N\cdot I\cdot L}=\frac{\sum _{1}^{N}{\int }_{0}^{\tau }{\int }_{0}^{{A}_{0}}{C}_{D}\rho {|V|}^{3}dAdt}{N\cdot I\cdot L}$$

where *N* is the annual number of TCs reaching the intensity of gale-fore winds; ${L}_{i}$, ${I}_{i}$, and ${S}_{i}$ are the weighted TC lifetime, and the TC intensity in the wind speed cube and the TC size in the area of the gale-force winds of the storm *i*, respectively; *L*, *I*, and *S* are the annual-averaged weighted lifetime, intensity, and size, respectively; ${V}_{\max }$ is the MWS, and ${V}_{{\rm{smax}}}$ is the lifetime peak intensity of the storm *i*; ${C}_{D}$ is the surface drag coefficient, $\rho$ is the surface air density, and $|V|$ is the magnitude of the surface wind.

Based on Eqs (1)–(4), the annually accumulated PDS is *N* × *L* × *I* × *S*, which follows the relation ln (PDS) = ln (*N*) + ln (*L*) + ln (*I*) + ln (*S*). Thus, the contributions of *N*, *L*, *I*, and *S* to PDS can be estimated. The results are shown in Table 1.

## Electronic supplementary material

Supplementary information
