# Supplementary material for: Impact of Ocean Warming on Tropical Cyclone Size and Its Destructiveness
Source: Sci Rep. 2017 Aug 15;7:8154. doi: 10.1038/s41598-017-08533-6 (PMC5557849; doi:10.1038/s41598-017-08533-6)
Supplement: Supplementary file 1 — Supplementary information [file 41598_2017_8533_MOESM1_ESM.doc]

Supplementary information for *Scientific Reports*

**Impact of Ocean Warming on Tropical Cyclone Size and Its Destructiveness**

Yuan Sun^1,2,+^, Zhong Zhong^2,+^, Tim Li^1,3,*^, Lan Yi^4^, Yijia Hu^2^, Hongchao Wan^5^, Haishan Chen^1^, Qianfeng Liao^2^, Chen Ma^1^, Qihua Li^2^

^1^International Laboratory on Climate and Environment Change, and Key Laboratory of Meteorological Disaster of Ministry of Education, Nanjing University of Information Science and Technology, Nanjing 210044, China

^2^College of Meteorology and Oceanography, National University of Defense Technology, Changsha 410073, China

^3^IPRC and Department of Atmospheric Sciences, University of Hawaii at Manoa, Honolulu, Hawaii 96815, USA

^4^Chinese Academy of Meteorological Sciences, Beijing 100081, China

^5^Meteorological Observatory of Hankou Station, Wuhan 430000, China

^*^Corresponding author. Email: [timli@hawaii.edu](mailto:timli@hawaii.edu)

^+^These authors contributed equally to this work

**Model design of the extra climate simulations considering the change of atmospheric lateral boundary conditions with SST warming.** To investigate the possible impact of atmospheric lateral boundary conditions on the main results, we have conducted three extra sets of 5-month (May-November) ensemble simulations with different SST patterns (i.e., CTRL_extra_, E_all+1-extra_, and E_all+2-extra_) over the WNP and NA respectively. The initial and lateral boundary conditions are derived from parallel runs of a global WRF model at a coarse grid (grid spacing of 1°) (i.e., CTRL_global_, E_all+1-global_, and E_all+2-global_). The initial conditions for the global climate simulations were obtained from the monthly mean 1° × 1° NCEP-FNL analysis data averaged from 2001 to 2010. The new sets of finer-grid (grid spacing of 0.2°) regional climate simulations are referred to as CTRL_extra_, E_all+1-extra_, and E_all+2-extra_. All parameterization schemes and the model domains of these extra simulations are same as the original simulations with the present-day atmospheric conditions as lateral boundary conditions.

**Table S1. Welch’s *t*-test statistics to test whether the impacts of SST warming on PDI and PDS are significant.** *p*-value is the probability of observing a test statistic as extreme as, or more extreme than, the observed value under the null hypothesis. If *p*-value < 0.05, the results would be deemed significant (in bold).

| Factors | *p*-value | | | | | | |
| --- | --- | --- | --- | --- | --- | --- | --- |
|  | WNP | | |  | NA | | |
|  | E_FSST_ | E_all+1_ | E_all+2_ |  | E_FSST_ | E_all+1_ | E_all+2_ |
| AGW | **1.4×10^-4^** | **2.0×10^-3^** | **4.1×10^-6^** |  | **4.5×10^-2^** | 5.2×10^-1^ | **1.4×10^-3^** |
| PDI | **9.8×10^-6^** | **1.1×10^-4^** | **7.5×10^-13^** |  | **8.5×10^-4^** | **1.0×10^-4^** | **4.6×10^-7^** |
| PDS | **2.3×10^-4^** | **1.1×10^-3^** | **1.7×10^-6^** |  | **1.7×10^-3^** | **5.5×10^-6^** | **8.4×10^-7^** |

**Table S2.** **Summary of results of the control experiments and sensitivity experiments for climate simulations over the WNP and the NA:** 10-year (2001-2010) averaged area of gale-force winds (AGW; 10^5^ km^2^), PDI (10^11^ m^3^ s^-2^) and PDS (10^18^ kg m^2^ s^-2^) of TCs.

| Factors | Basin | | | | | | | | |
| --- | --- | --- | --- | --- | --- | --- | --- | --- | --- |
|  | WNP | | | |  | NA | | | |
|  | CTRL | E_FSST_ | E_all+1_ | E_all+2_ |  | CTRL | E_FSST_ | E_all+1_ | E_all+2_ |
| AGW | 0.46 | 1.94 | 1.05 | 3.63 |  | 1.33 | 2.30 | 1.58 | 2.59 |
| PDI | 18.1 | 110.1 | 69.9 | 137.7 |  | 9.0 | 56.2 | 31.5 | 66.5 |
| PDS | 11.2 | 110.0 | 49.6 | 215.0 |  | 16.1 | 86.0 | 41.7 | 102.9 |

**Table S3.** **Summary of experiment designs for the climate simulations and TC cases simulations.**

|  | **Climate simulations** | | **TC cases simulations** | |
| --- | --- | --- | --- | --- |
|  | WNP | NA | Songda (2004) | Bill (2009) |
| Domain dimensions | 400×300 grids, centered at 140°E, 30°N | 400×300 grids, centered at -60°W, 30°N | Outer domain: 211×205 grids, centered at 137.5°E, 28°N  Moving inner domain: 200×200 grids | Outer domain: 249×299 grids, centered at 51.5°W, 30°N  Moving inner domain: 200×200 grids |
| Resolutions | 0.2° | | Outer domain: 20 km  Inner Domain: 4 km | |
| Simulation time | 10-year (2001-2010) typhoon season (from May 1 to November 1) | | Outer domain: from 0000 UTC 26 August to 0600 UTC 7 September 2004  Inner domain: from 1800 UTC 30 August to 0600 UTC 7 September 2004 | Outer domain: from 0000 UTC 16 August to 0600 UTC 24 August 2009  Inner domain: from 1800 UTC 16 August to 0600 UTC 24 August 2009 |
| Microphysics | WSM 3-class simple ice scheme | | WSM 3-class simple ice scheme | |
| Cumulus parameterization | Grell-Devenyi ensemble scheme | | Kain-Fritsch (new Eta) scheme | |
| Boundary layer | YSU scheme | | Mellor-Yamada-Janjic (Eta) TKE scheme | YSU scheme |
| Radiation | Longwave: RRTM scheme  Shortwave: Dudhia scheme | | Longwave: RRTM scheme  Shortwave: Goddard scheme | Longwave: RRTM scheme  Shortwave: Dudhia scheme |

**Table S4.** **Summary of the control and sensitivity experiments for the cases of Songda (2004) in the WNP and Bill (2009) in the NA:** the variable *r* is the radial distance from the storm center; ΔSST is the artificial modification of SST; MSLP_min_ and V_max_ are the lifetime minimum MSLP and lifetime maximum wind speed at 10-m for each simulation; RMW_ave_ is the RMW at 10-m averaged over the simulation period (from 1800 UTC 30 August to 0600 UTC 7 September 2004 for Songda case, and from 1800 UTC 16 August to 0600 UTC 24 August 2009 for Bill case).

| **Exp.** | **Modification to the underlying SST** | **MSLP_min_(hPa)**  **Songda/Bill** | **V_max_(m s^-1^)**  **Songda/Bill** | **RMW_ave_ (km)**  **Songda/Bill** |
| --- | --- | --- | --- | --- |
| CTRL | Control experiment | 928.2/958.3 | 53.8/48.3 | 61.5/62.4 |
| E_FSST_ | Future SST experiment | 896.8/903.2 | 62.0/64.7 | 89.2/78.4 |
| E_40+1_ | ΔSST = 1°C for *r*≤40 km, and linearly increases to 0°C at *r*=60 km | 929.7/956.7 | 53.4/46.2 | 59.5/59.8 |
| E_100+1_ | ΔSST = 1°C for *r*≤100 km, and linearly increases to 0°C at *r*=120 km | 916.0/954.7 | 56.8/51.5 | 54.4/54.2 |
| E_200+1_ | ΔSST = 1°C for *r*≤200 km, and linearly increases to 0°C at *r*=220 km | 913.7/955.2 | 57.9/49.9 | 57.2/59.7 |
| E_300+1_ | ΔSST = 1°C for *r*≤300 km, and linearly increases to 0°C at *r*=320 km | 913.6/960.7 | 58.1/49.3 | 59.8/67.8 |
| E_all+1_ | ΔSST = 1°C for all the simulation domain | 922.1/954.0 | 55.0/49.5 | 73.0/63.6 |
| E_40+2_ | ΔSST = 2°C for *r*≤40 km, and linearly increases to 0°C at *r*=60 km | 925.0/949.5 | 54.6/51.2 | 57.8/56.9 |
| E_100+2_ | ΔSST = 2°C for *r*≤100 km, and linearly increases to 0°C at *r*=120 km | 904.0/944.3 | 58.8/55.1 | 48.3/48.9 |
| E_200+2_ | ΔSST = 2°C for *r*≤200 km, and linearly increases to 0°C at *r*=220 km | 899.7/950.7 | 62.4/52.6 | 53.9/60.9 |
| E_300+2_ | ΔSST = 2°C for *r*≤300 km, and linearly increases to 0°C at *r*=320 km | 905.5/946.4 | 60.5/53.9 | 62.6/64.0 |
| E_all+2_ | ΔSST = 2°C for all the simulation domain | 906.9/937.5 | 60.5/53.9 | 98.4/66.9 |
| E_all+4_ | ΔSST = 4°C for all the simulation domain | 839.2/888.9 | 61.4/62.5 | 77.8/58.6 |
| E_all+6_ | ΔSST = 6°C for all the simulation domain | 788.3/861.0 | 65.7/69.8 | 106.4/73.2 |

**Table S5.** **As in Table S2, but for the extra climate simulations considering the change of atmospheric conditions with SST:** Annual area of gale-force winds (10^5^ km^2^), PDI (10^11^ m^3^ s^-2^) and PDS (10^18^ kg m^2^ s^-2^) of TCs.

| Factors | Basin | | | | | | |
| --- | --- | --- | --- | --- | --- | --- | --- |
|  | WNP | | |  | NA | | |
|  | CTRL | E_all+1_ | E_all+2_ |  | CTRL | E_all+1_ | E_all+2_ |
| AGW | 2.38 | 3.48 | 10.69 |  | 1.33 | 9.91 | 15.03 |
| PDI | 27.0 | 37.8 | 34.2 |  | 5.4 | 4.9 | 17.1 |
| PDS | 49.9 | 73.4 | 111.1 |  | 10.3 | 39.8 | 116.9 |


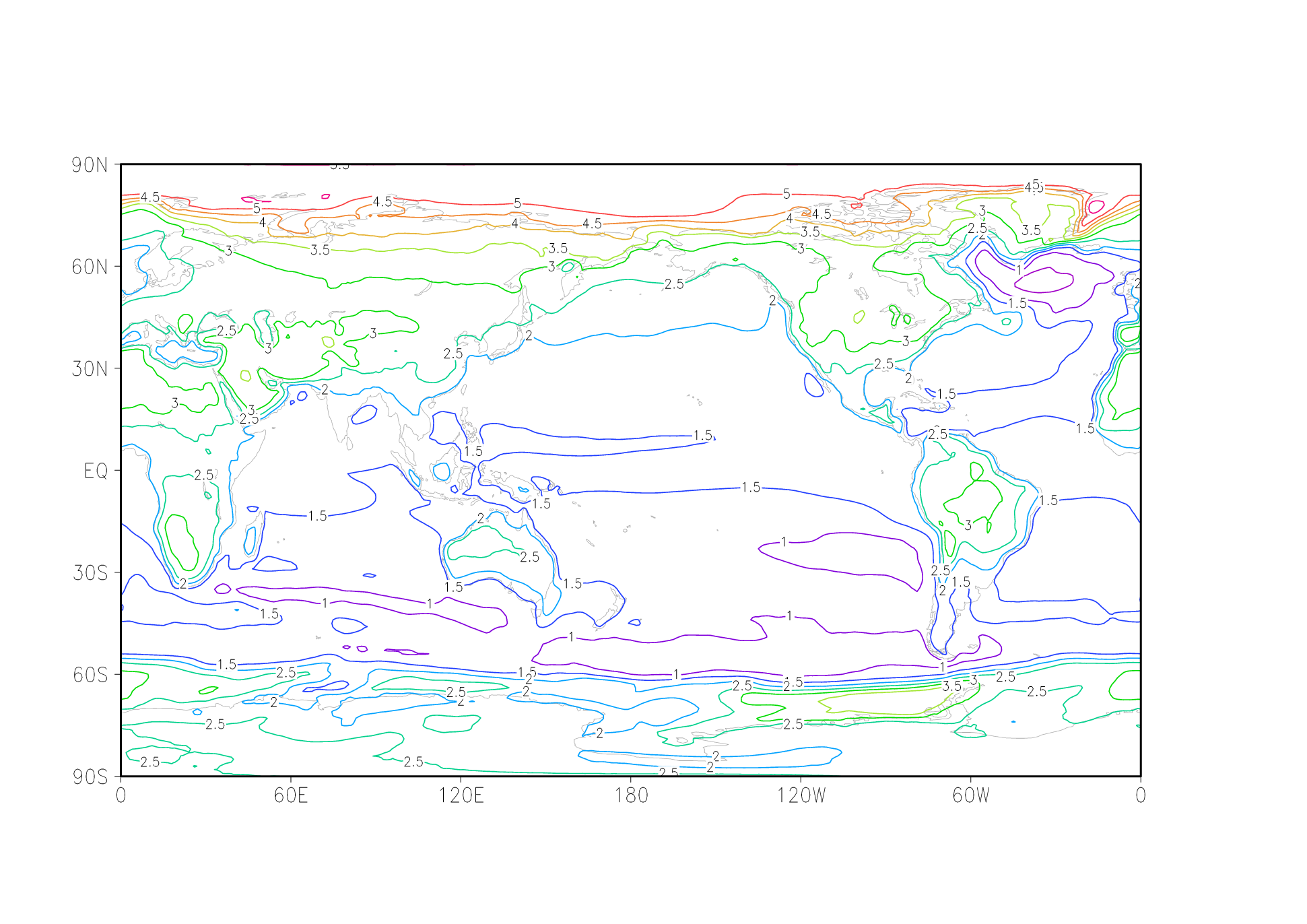


**Figure S1. The projected multi-model mean global SST anomalies from 33 CMIP-5 coupled models averaged during the TC season of July-October, based on a doubling of CO2 experiment.** The map is created by the Grid Analysis and Display System Version 2.0 (GrADS V2.0, http://grads.iges.org/grads/).


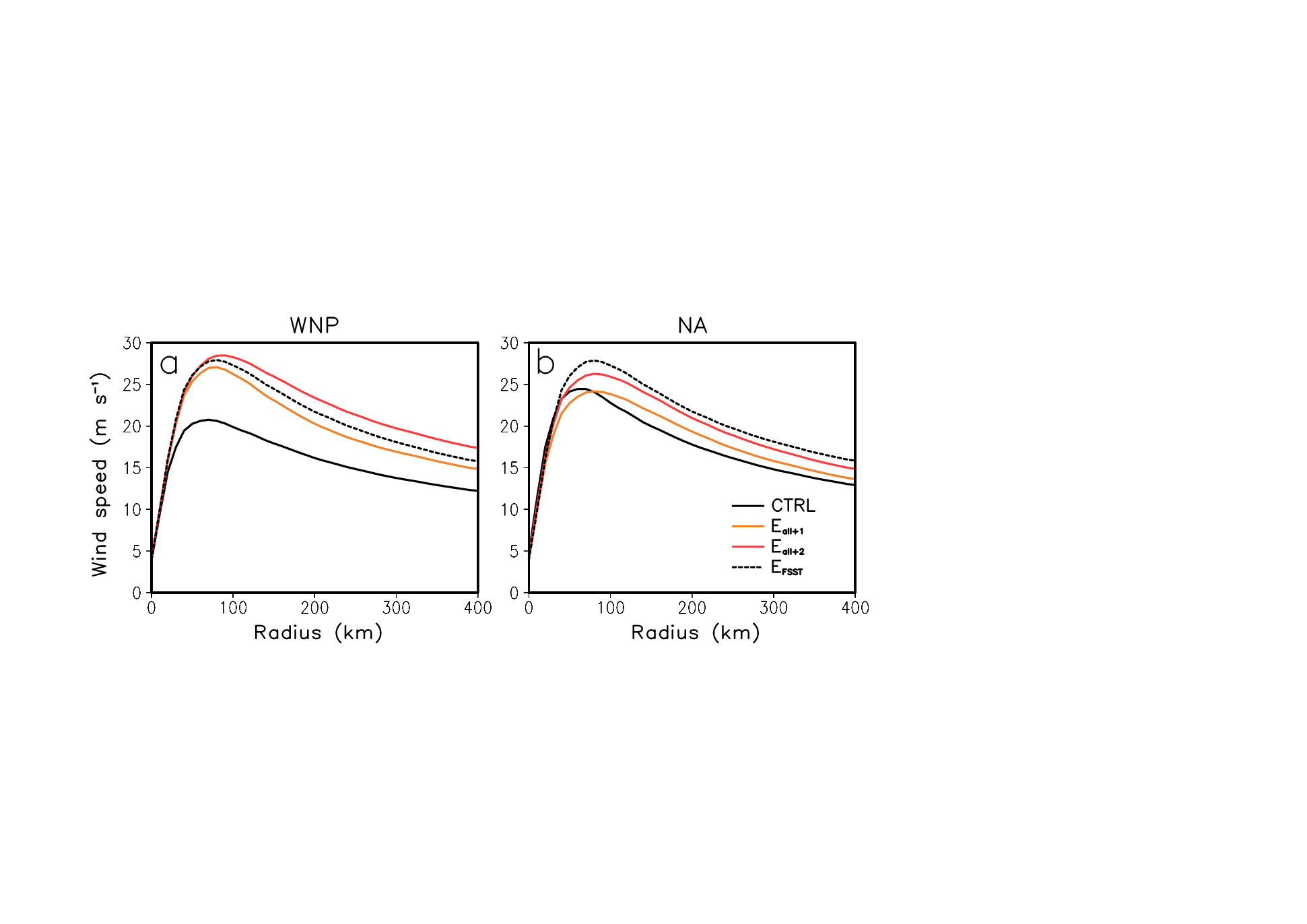


**Figure S2. Radial distributions of the** **azimuthal mean wind speed at 10-m** **averaged during the TC mature stage over the WNP and the NA.** TC mature stage is defined as the period when the maximum wind speed at 10-m (V_max_) is close to its lifetime maximum wind speed at 10-m (V_smax_), i.e., |V_max_-V_smax_| $\leq$3 m s^-1^.


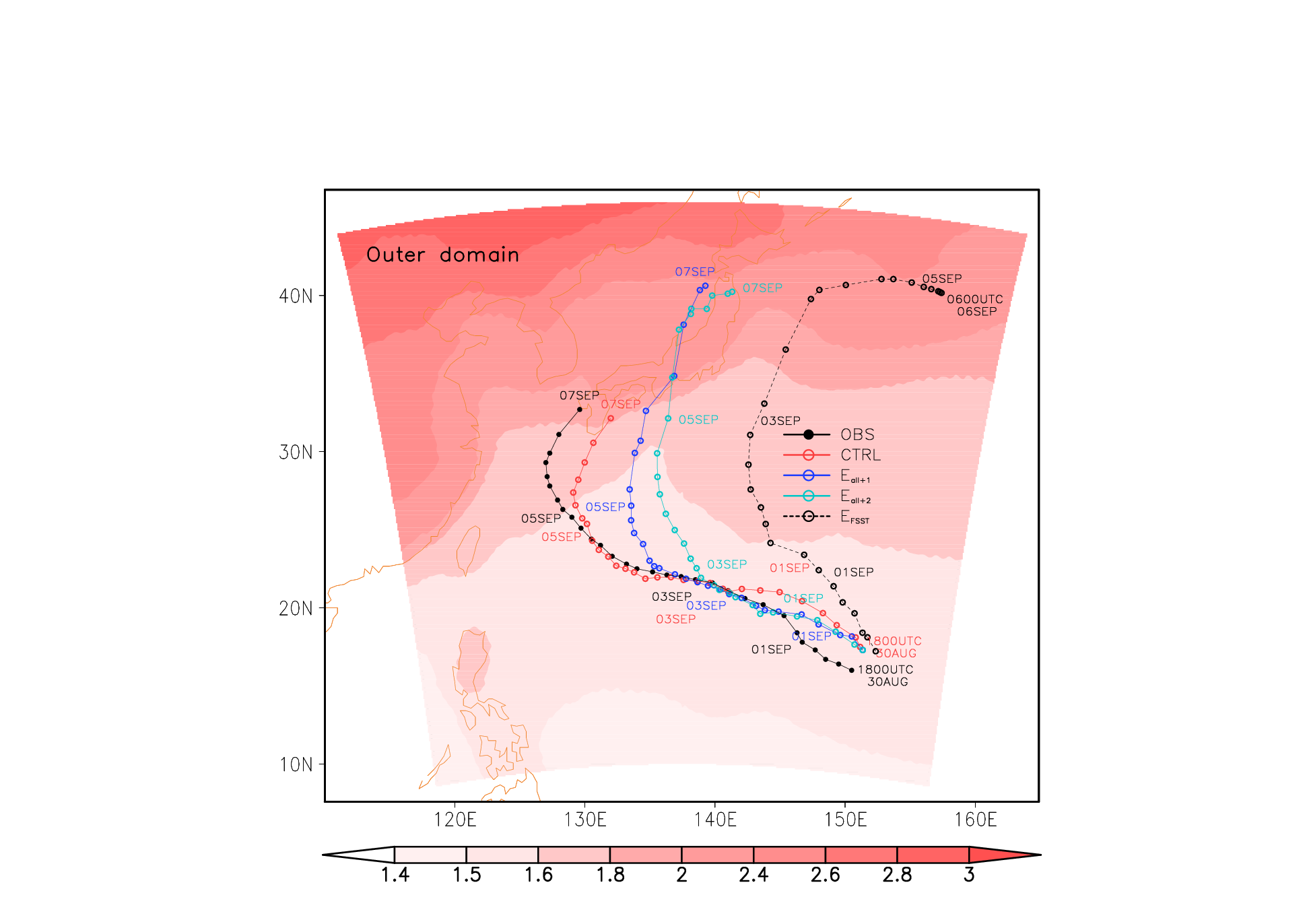


**Figure S3. The outer domain of the model and the simulated storm track superimposed on the observed best track at 6-h intervals for Songda (2004) case.** The background shading is the August-averaged SST anomalies in E_FSST_. The map is created by the Grid Analysis and Display System Version 2.0 (GrADS V2.0, http://grads.iges.org/grads/).


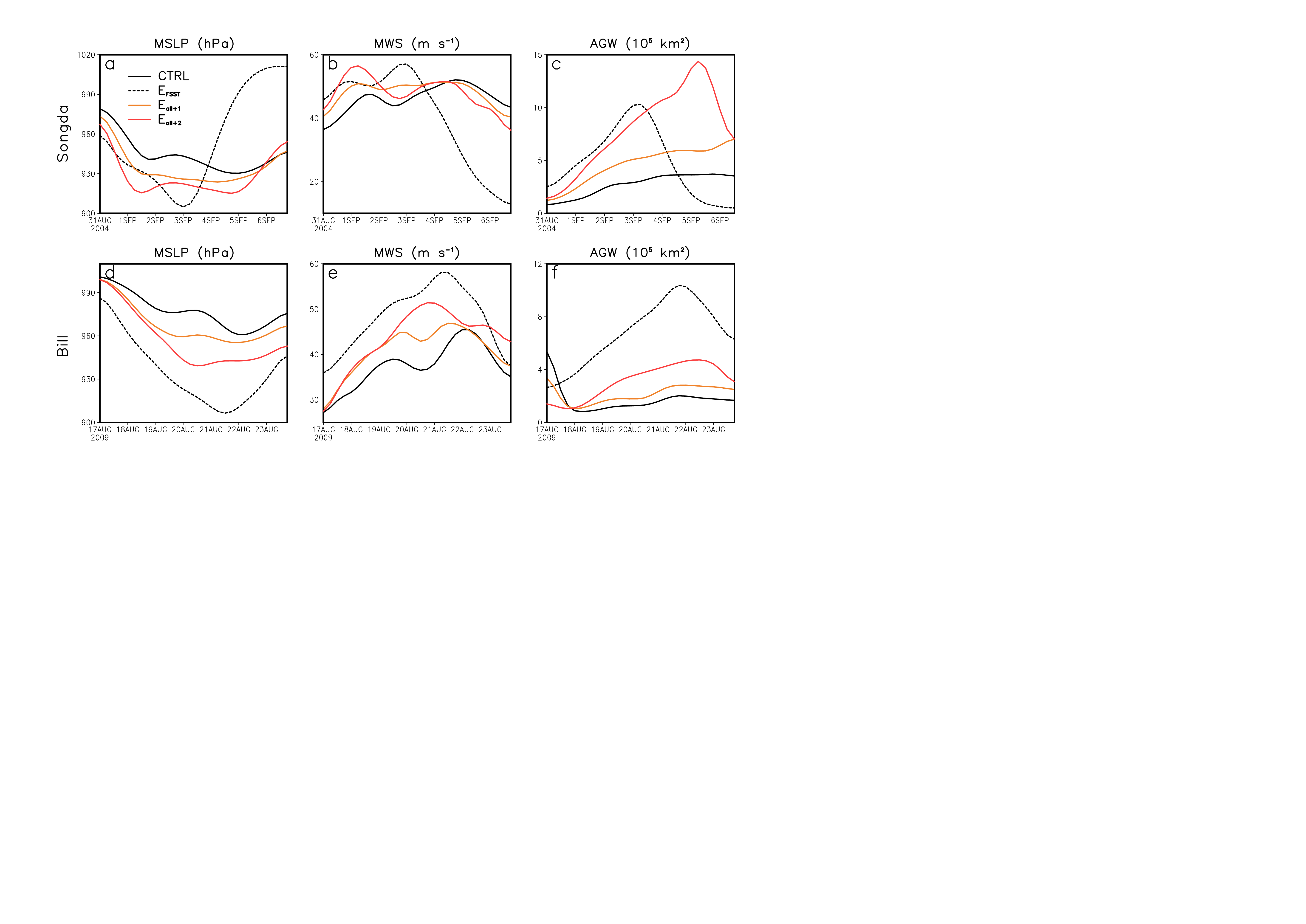


**Figure S4. Temporal evolutions of MSLP, MWS, and AGW in the sensitivity experiments for the Songda (2004) and Bill (2009) cases.**


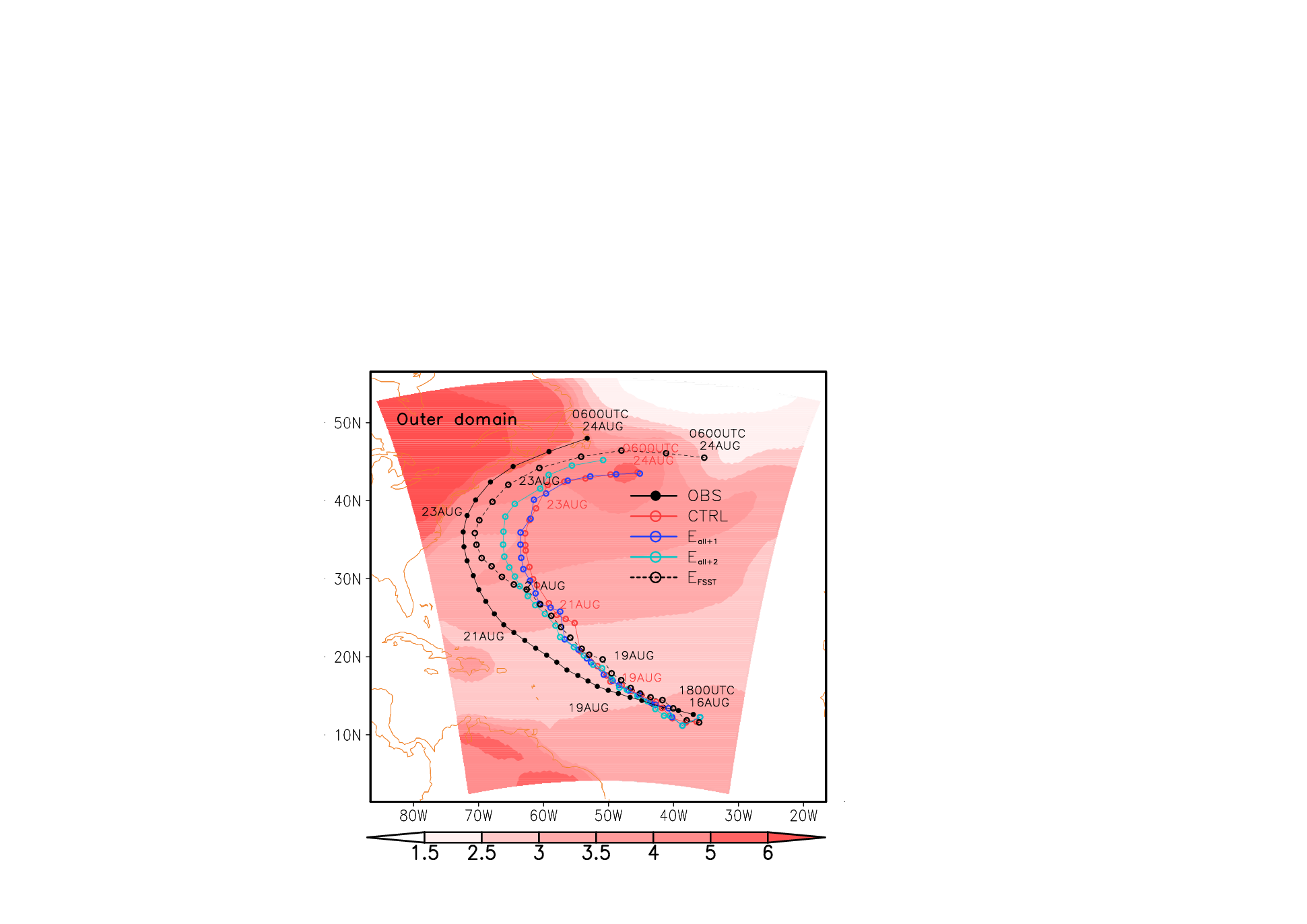


**Figure S5. The outer domain of the model and the simulated storm track superimposed on the observed best track at 6-h intervals for Bill (2009) case.** The background shading is the August-averaged SST anomalies in E_FSST_. The map is created by the Grid Analysis and Display System Version 2.0 (GrADS V2.0, http://grads.iges.org/grads/).


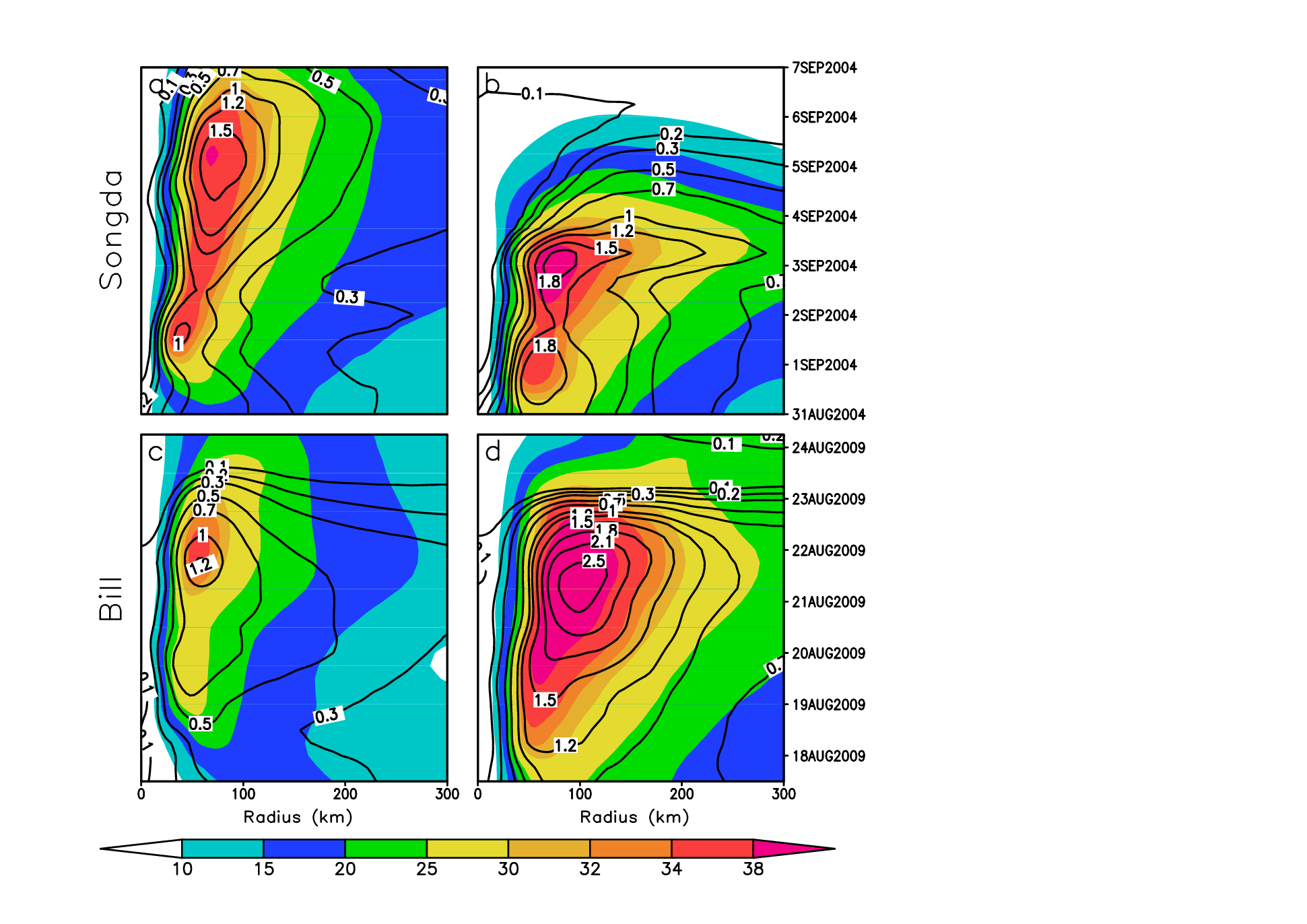


**Figure S6. Hovmöller diagrams of the azimuthal-averaged surface wind speed (shaded; m s^-1^) and SEF (contour; 10^3^W m^-2^).** (a) For the CTRL experiment of Songda case; (b) for the E_FSST_ experiment of Songda case; (c) for the CTRL experiment of Bill case; (d) for the E_FSST_ experiment of Bill case.


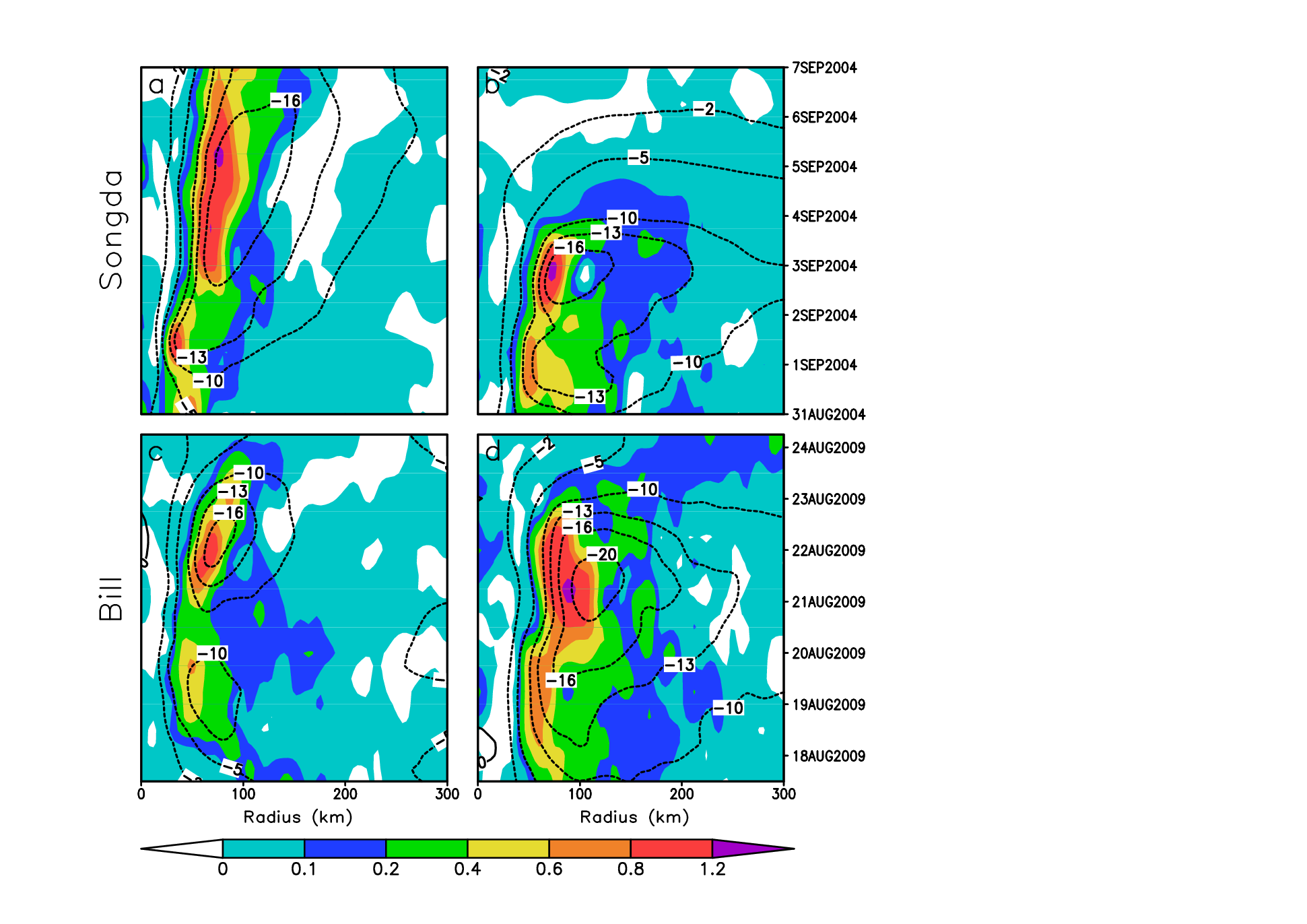


**Figure S7. Hovmöller diagrams of the azimuthal-averaged vertical motion at 700 hPa (shaded; m s^-1^) and the radial wind speed at 10 m (contour; m s^-1^)** (a) for the CTRL experiment of Songda case; (b) for the E_FSST_ experiment of Songda case; (c) for the CTRL experiment of Bill case; (d) for the E_FSST_ experiment of Bill case.


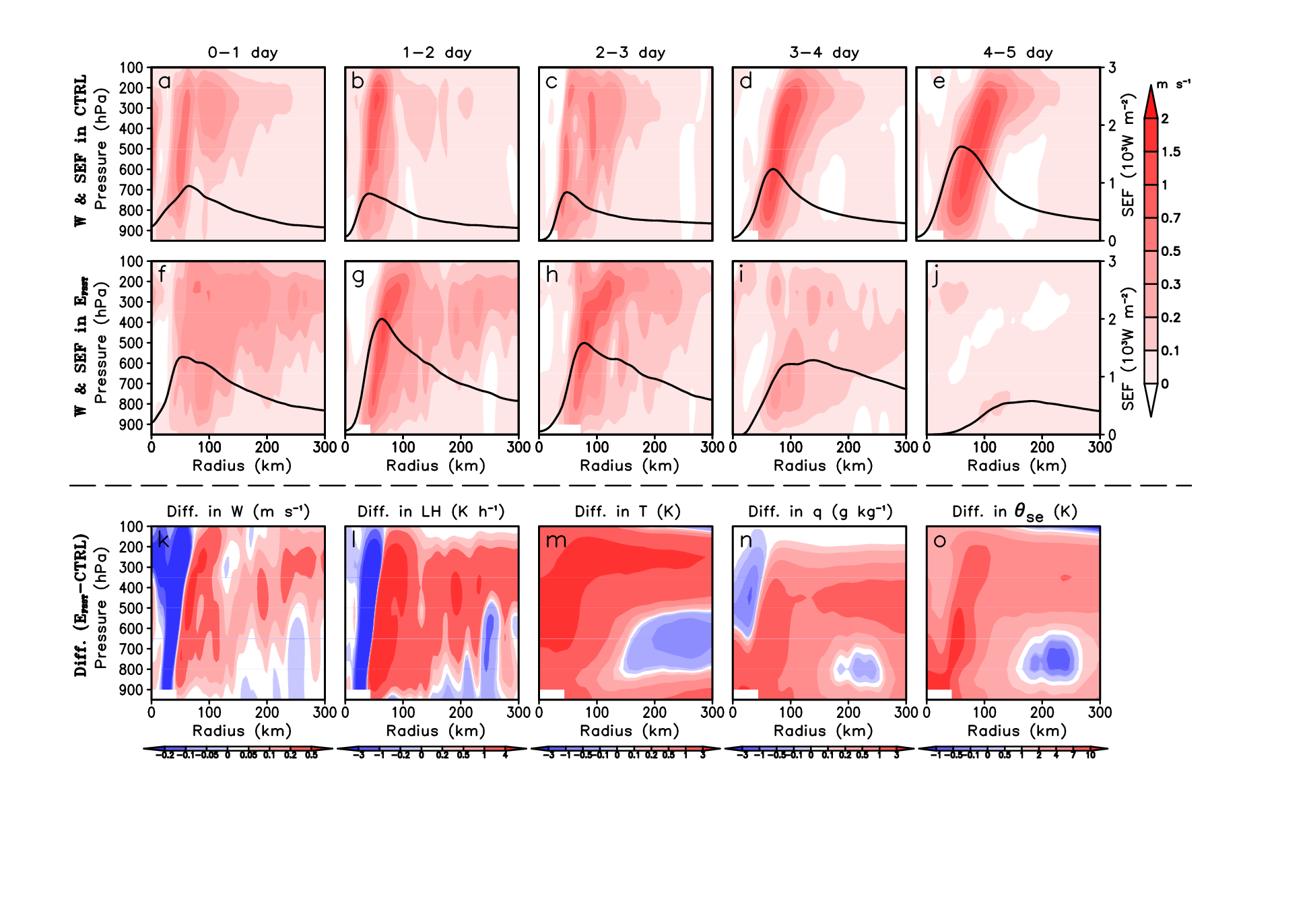


**Figure S8. Impact of SST warming on the time- and azimuthal-averaged structure of TC for Songda (2004) case. (a-j)** Azimuthal-cross sections of the vertical velocity (shaded; m s^-1^) and surface entropy flux (contour; 10^3^W m^-2^) in the CTRL (**a**-**e**) and E_FSST_ (**f**-**j**) at various periods; (**k**-**p**) Azimuthal-cross sections of the 3-4 day time-averaged differences in the vertical velocity (m s^-1^), latent heating (LH; K h^-1^), temperature (T; K), water vapor mixing ratio (q; g kg^-1^) and the potential pseudo-equivalent temperature (𝜃_se_; K) between E_FSST_ and CTRL experiments.


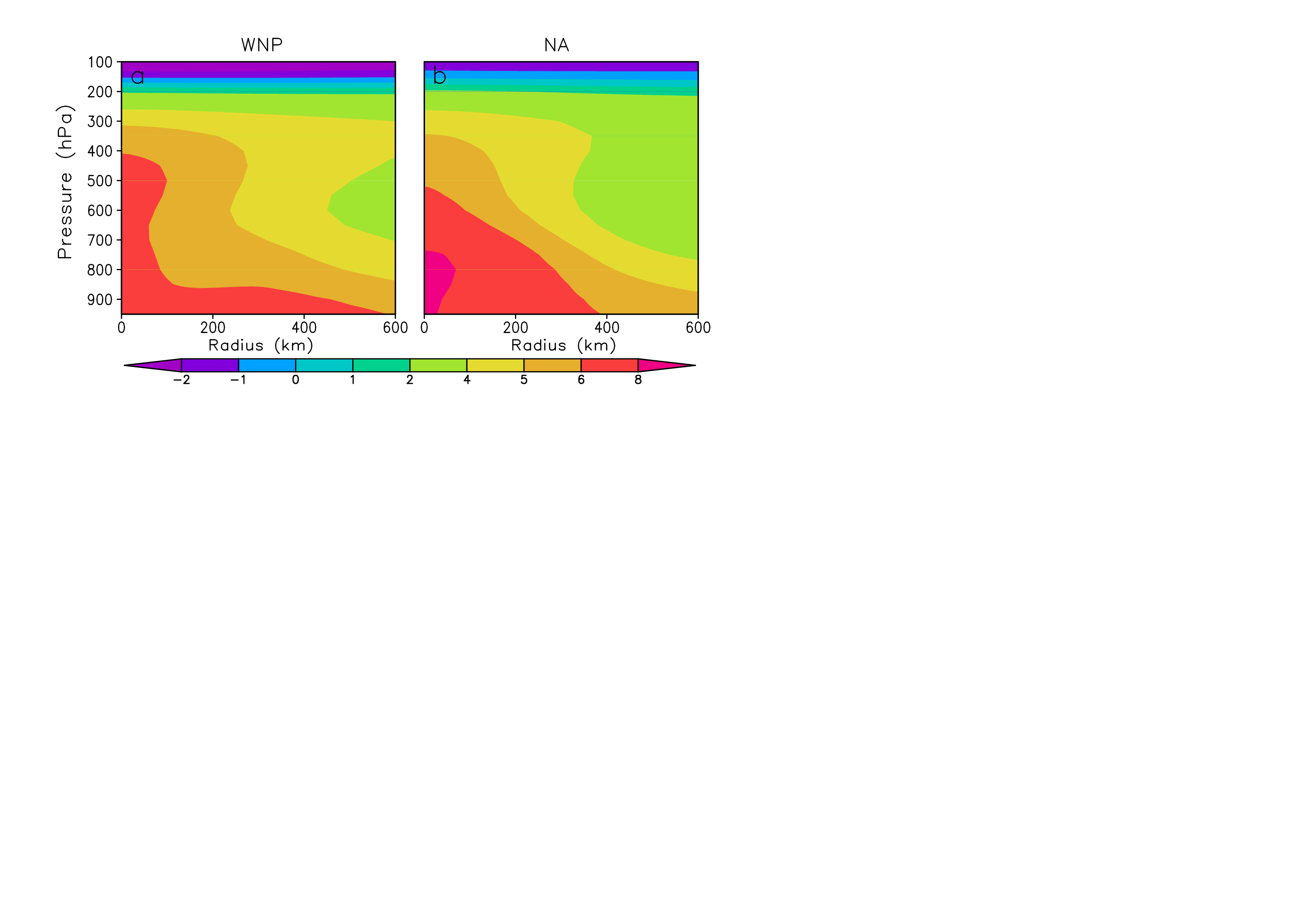


**Figure S9. Time- and azimuthal-averaged potential pseudo-equivalent temperature (𝜃_se_) difference between E_all+1_ and CTRL experiments for the TCs over the WNP and the NA.**





**Figure S10. Time- and domain-averaged difference in 200-hPa temperature between the sensitivity experiments and the CTRL experiment during the typhoon season (May-November) over the main TC genesis area in the WNP and NA (120****°E-160°E, 10°N-30°N for the WNP; 60°W-20°W, 5°N-25°N for the NA) for the original and extra climate simulations.**


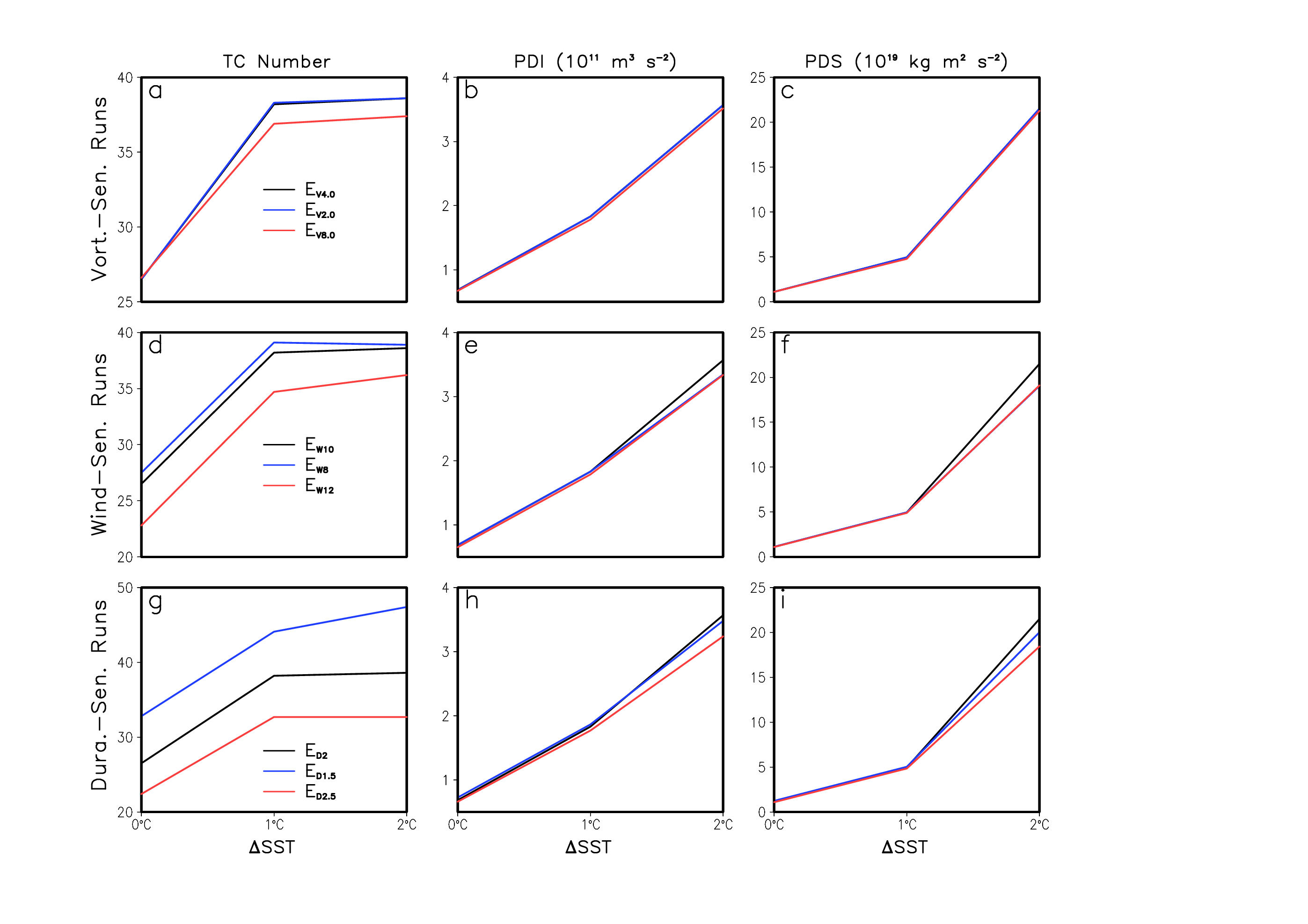


**Figure S11. Sensitivity of annual TC number, PDI (10^11^ m^3^ s^-2^) and PDS (10^19^ kg m^2^ s^-2^) during the typhoon season (May-November) to the three vortex criteria over the WNP in the climate simulations.** The annual PDI and PDS are averaged over 10 years (2001-2010). **(a-c)** The upper panels represent the results of the sensitivity experiments with different maximum vorticities [e.g., E_V4.0_ is the suite of the experiments (i.e., CTRL, E_all+1_, and E_all+2_) with the maximum relative vorticity at 850 hPa exceeds 4.0×10^-5^ s^-1^]; **(d-f)** the middle panels represent the results of the experiments with different maximum wind speed [e.g., E_W10_ is the suite of the experiments (i.e., CTRL, E_all+1_, and E_all+2_) with the maximum wind speed at 10-m is greater 10 m s^-1^]; **(g-i)** the bottom panels represent the results of the experiments with different durations [e.g., E_D2_ is the suite of the experiments (i.e., CTRL, E_all+1_, and E_all+2_) with the TC lifetime at least 2 days).


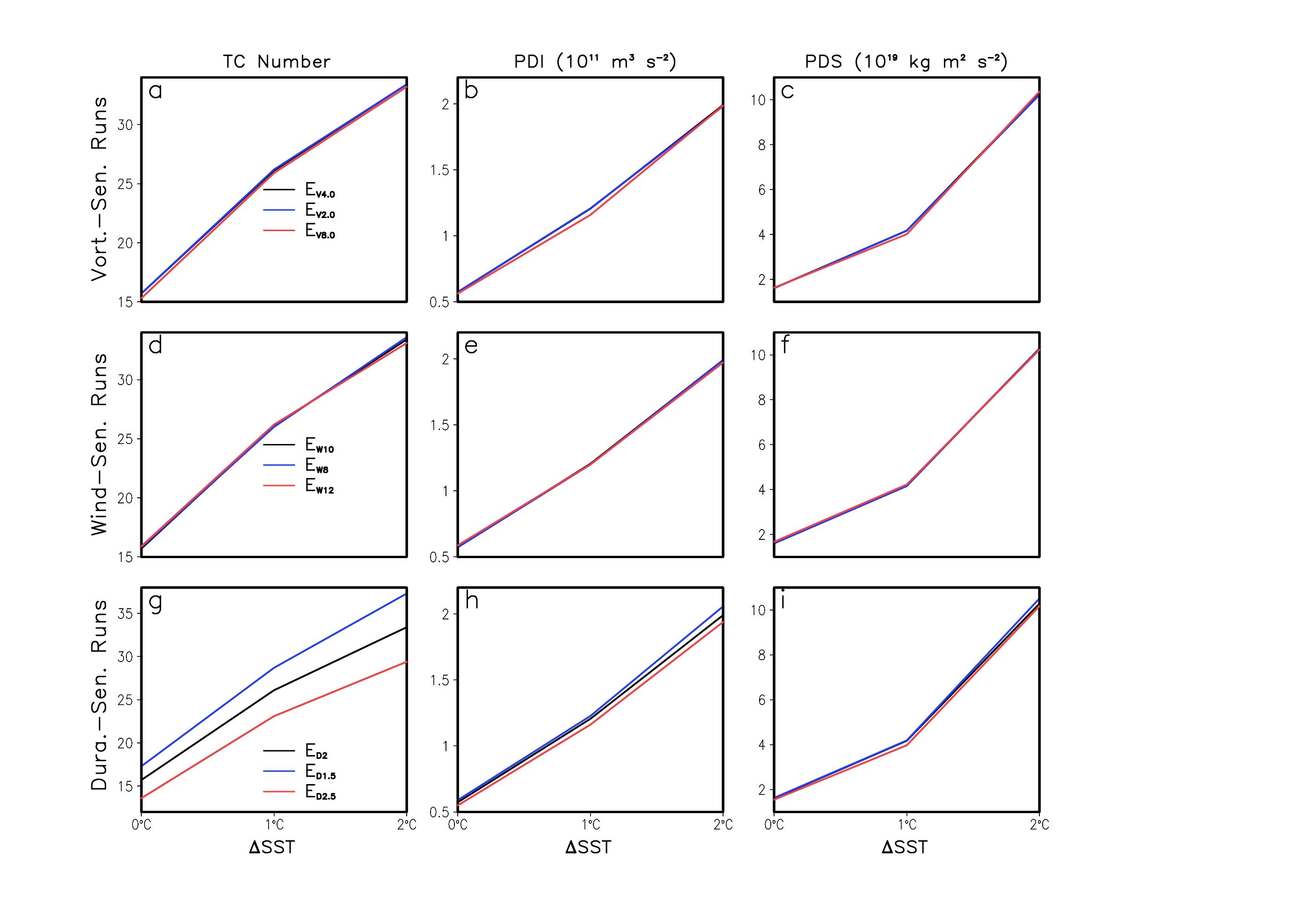


**Figure S12. As in Figure S11, but for the climate simulations over the NA.**
